# Experimental and Mathematical-Modeling Characterization of *Trypanosoma cruzi* Epimastigote Motility

**DOI:** 10.1371/journal.pone.0142478

**Published:** 2015-11-06

**Authors:** Eduardo Sosa-Hernández, Gilberto Ballesteros-Rodea, Jorge A. Arias-del-Angel, Diego Dévora-Canales, Rebeca G. Manning-Cela, Jesús Santana-Solano, Moisés Santillán

**Affiliations:** 1 Unidad Monterrey, Centro de Investigación y de Estudios Avanzados del IPN, Apodaca NL, México; 2 Facultad de Agronomía y Veterinaria, Universidad Autónoma de San Luis Potosí, SLP, México; 3 Depto. de Biomedicina Molecular, Centro de Investigación y de Estudios Avanzados del IPN, México DF, México; Pennsylvania State Hershey College of Medicine, UNITED STATES

## Abstract

The present work is aimed at characterizing the motility of parasite *T. cruzi* in its epimastigote form. To that end, we recorded the trajectories of two strains of this parasite (a wild-type strain and a stable transfected strain, which contains an ectopic copy of *LYT1* gene and whose motility is known to be affected). We further extracted parasite trajectories from the recorded videos, and statistically analysed the following trajectory-step features: step length, angular change of direction, longitudinal and transverse displacements with respect to the previous step, and mean square displacement. Based on the resulting observations, we developed a mathematical model to simulate parasite trajectories. The fact that the model predictions closely match most of the experimentally observed parasite-trajectory characteristics, allows us to conclude that the model is an accurate description of *T. cruzi* motility.

## Introduction

*Trypanosoma cruzi* is a flagellated protozoan that causes Chagas disease, also known as American Trypanosomiasis. This disease affects approximately 8-10 million people, while more than 20 million people in Latin America are at risk of contracting it [1].

The parasite *T. cruzi* presents a digenetic life cycle, alternating between blood-sucking triatome and mammals. In the mammalian bloodstream, the parasite can be found in the trypomastigote form. When an insect vector bites a mammal and ingests trypomastigotes, they transform epimastigotes. Later, in the insect intestines, epimastigotes differentiate into metacyclic trypomastigotes. When the insect bites a healthy mammal, these trypomastigotes are excreted in the insect feces and they enter the mammal bloodstream, usually through some skin injury. Once in the vertebrate host, metacyclic trypomastigotes infect different cells such as: fibroblasts, macrophages, and epithelial cells [2]. Within the infected cells, metacyclic trypomastigotes transform into amastigotes, which reproduce before being transformed into bloodstream trypomastigotes. Finally, the infected cells are lysed and bloodstream trypomastigotes are released into the vertebrate bloodstream [3].

During the life cycle of *T. cruzi*, two developmental stages of the parasite have flagellum: the epimastigote and the trypomastigote. The flagellum has important functions like: sensitivity, reproduction and motility [4]. Moreover, it is suspected that besides allowing the parasite to move, *T. cruzi* motility is important for both the reproduction and infection processes [5, 6]. Indeed, recent studies [7, 8] have proved that various factors that affect *T. cruzi* motility also inhibit infection.

The cellular and molecular aspects of *T. cruzi* are the subject of active research [9–11]. However, despite its importance, parasite motility has been mostly disregarded. A previous report by our group [12] suggests that *T. cruzi* movements involve a propulsion term, as well as non-propelling fluctuations similar to Brownian motion. Similar studies on a related parasite, *T. brucei*, studied with more detail its motility. In particular, they demonstrated that such parasite trajectories can be classified as either tumbling or persistent [13, 14]. However, very little is known about the way *T. cruzi* parasites move, what factors affect their movement, etc. The present paper is aimed at partially filling this void. To do this, we take existent methodology, widely employed to study other microorganisms’ motility [13–19], and adapt it to investigate and characterize *T. cruzi* epimastigote motility. We employ a wild-type strain, as well as a stable transfected parasite strain, which contains an ectopic copy of *LYT1* gene, as a mobility defective control. Although the causes of the reduced motility of this transgenic *T. cruzi* strain are not fully understood yet, there is evidence that an LYT1 isoform localizes in the mitochondrial kinetoplast zonal region [20]. Additionally, an MS-MS approached study [21] described multiple protein interaction associations of LYT1, including with dynein and both alpha-and beta-tubilins. Hence, it is possible that the downstream effects of *LYT1* over-expression, with respect to parasite motility, are due to the iteraction of LYT1 with the above referred proteins, which are known to be involved in the parasite vesicular traffic. Since this mechanism is responsible for the transport of molecules that are essential for the growth and good performance of the flagellum, its alteration could explain the observed deleterious phenotype [22].

## Materials and Methods

### Parasite strains

The parasite strains employed in this work were: epimastigotes of *T. cruzi* CL-Brener strain, a clone obtained from the CL strain isolated from Triatoma infestans in Rio Grande, Brazil [23], and a transgenic *T. cruzi* strain that was engineered by ourselves as detailed in [20]. Although we were able to engineer strains that overexpress different combinations of the two isoforms of LYT1 protein, we chose to work with the one that overexpresses both isoforms. The reason being that, when contrasted with the wild type strain, the chosen transgenic strain presents the most noticeable effects regarding motility.

### Culture media

Wild type (WT) and transgenic *T. cruzi* CL-Brener strains were cultured in liver infusion tryptose LIT medium, supplemented with 10% fetal bovine serum (FBS), 0.5% penicillin (10,000 IU) / streptomycin (10.000 *μ*g), and 1% of hemin (5 mg/ml), at 28°C. The parasites were grown in homogeneous conditions and were sampled for use in experiments while in the logarithmic growth stage at 48 hours. The stock cultures were grown every 4 days using 1 × 10^6^ parasites as an initial inoculum to guarantee the homogeneity and active motility of the parasites.

### Sampling preparation and video recording

Culture medium, containing *T. cruzi* epimastigotes, was mixed with latex beads of 10 *μ*m diameter. A 10 *μ*l drop of this mixture was placed on a slide, covered with a coverslip, and sealed with poly-dimethyl-siloxane (PDMS). Slides and coverslips were previously treated with pvp-40 on miliQ water solution at 0.05%*w*/*v* to prevent parasites to attach to glass. The latex beads allowed to keep a constant 10 *μ*m plate separation, and this in turn made the parasites follow quasi 2-dimensional trajectories. Videos were recorded at 30 frames per second by means of a CCD camera connected to a phase contrast microscope Olympus (BX51), with a 40X magnification objective. The temperature during experiments was 25°C.

### Image processing for obtaining parasite trajectories

Videos for each one of the studied *T. cruzi* strains were analysed with Matlab. To obtain parasite trajectories, we analysed all the individual frames in each video. In every frame, the parasites were spotted as dark or white figures with a gray background. The positions of white and dark figures were computed by means of image processing tools like derivative, dilatation, and threshold binarization. Finally, we located the centroid of the resulting binary-image objects. Below, we give a detailed description of the whole process:

The first frame-analysis step was to compute the intensity gradient by applying an image filter over the *x* and *y* directions, with following differentiation kernels Eq (1):

$$\begin{array}{c} H_{\Delta x}=[\begin{array}{ccc} 1 & 0 & -1\\ 2 & 0 & -2\\ 1 & 0 & -1 \end{array}],\;H_{\Delta y}=[\begin{array}{ccc} 1 & 2 & 1\\ 0 & 0 & 0\\ -1 & -2 & -1 \end{array}]. \end{array}$$ (1)

Then, we reduced the gaps from parasite contours to get well defined objects. To increase image definition, the contours were dilated by 2 pixels from its base bright pixel. Full parasite contours made possible to reconstruct layers of same brightness for each parasite, making a top layer a good candidate to find the centroid location. Once we had the collection of all the parasite positions in all the frames of a video, we extracted the parasite trajectories via a well-known, specialized algorithm, originally implemented by David Grier, John Crocker, and Eric Weeks [24] to extract quantitative data from digitized video microscope images of colloidal suspensions. Essentially, this algorithm correlates positions from one image at time *t*_*i*_ to another image at time *t*_*i*−1_, and it does so by minimizing the error of all position combinations between the two. The parameters in this algorithm that need to be customized are listed below:

1. *Goodenough*, specifies the minimum trajectory length. In our case, parasites that were tracked for less than 10 frames were ignored.
2. *Memory*, enabled us to track parasites which temporarily disappear. For us, a parasite can be missing for up to 4 frames in a row, but if it reappears in the same location, it is still considered as the same parasite.
3. *Maxdisp*, is an estimate of the maximum distance that a parasite would move in a single time interval. We set the maximum parasite speed to 18 *pixels*.

### Trajectory analysis

After processing all the recorded videos, we obtained 147 trajectories (with an average length of 340 steps) for the wild-type strain, and 150 trajectories (with an average length of 2600 steps) for the genetically modified strain.

Instantaneous speed and velocity components in the horizontal and vertical directions were computed. This allowed us to visualize abnormal phenomena such as external flows and unexpected parasite activity level. So, when an external flow appears while analysing the trajectories of a given experiment, it is ignored. Similarly, exhaustion of nutrients is detected when the average instantaneous speed on sample decreases abnormally. When this happens, the corresponding experiments are ignored as well.

The parameters here proposed to characterize parasite motility are: the angular change of direction between consecutive steps (*θ*_*i*_), the components of the velocity at a given step that are longitudinal (*λ*_*i*_) and transverse (*τ*_*i*_) to the previous step velocity, and the mean square displacement (MSD). These parameters were computed, by means of the following equations, from the data generated by the image analysis process describe above:

$$\begin{array}{ccc} \vec{v_{i}} & = & \frac{\vec{r_{i+1}}-\vec{r_{i}}}{\Delta t}, \end{array}$$ (2)

$$\begin{array}{ccc} \theta_{i} & = & \sin^{-1}(\frac{{\vec{v}}_{i-1}\times {\vec{v}}_{i}}{|{\vec{v}}_{i-1}\left|\right|{\vec{v}}_{i}|}), \end{array}$$ (3)

$$\begin{array}{ccc} \lambda_{i} & = & {\vec{v}}_{i}\cdot \frac{{\vec{v}}_{i-1}}{|{\vec{v}}_{i-1}|}, \end{array}$$ (4)

$$\begin{array}{ccc} \tau_{i} & = & {\vec{v}}_{i}\cdot (\hat{k}\times \frac{{\vec{v}}_{i-1}}{|{\vec{v}}_{i-1}|}), \end{array}$$ (5)

$$\begin{array}{ccc} \left\langle \Delta {\vec{r}}^{2}\right\rangle & = & \langle {\left({\vec{r}}_{i+n\Delta t}-{\vec{r}}_{i}\right)}^{2}\rangle , \end{array}$$ (6)

where subindex *i* indicates the time stamp, Δ*t* = 1/30s is the time interval between consecutive frames, $\vec{r_{i}}=x_{i}\hat{i}+y_{i}\hat{j}+0\hat{k}$ represents the parasite position at time *i*, ${\vec{v}}_{i}$ is the instantaneous velocity, $\langle \Delta {\vec{r}}^{2}\rangle$ is the MSD, and 〈⋯〉 denotes ensemble average.

### Image processing for computing the flagellum beating frequency

In order to determine the flagellum beating frequency it was necessary to identify three vectors of reference for each trypanosoma: its centroid (${\vec{w}}_{c}$), the flagellum base point (${\vec{w}}_{i}$), and its tip (${\vec{w}}_{f}$). Then we measured, by means the following equation, the angle between the vectors $\vec{\alpha }={\vec{w}}_{f}-{\vec{w}}_{i}$ and $\vec{\beta }={\vec{w}}_{c}-{\vec{w}}_{i}$.

$$\begin{array}{c} \phi =\cos^{-1}(\frac{\vec{\alpha }\cdot \vec{\beta }}{|\vec{\alpha }\left|\right|\vec{\beta }|}). \end{array}$$ (7)

By following the time evolution of this angle, it was possible to determinate the flagellum beating frequency for each parasite. We did that by calculating the power spectra of the corresponding time series.

The procedure to determine the position of the three points of reference was as follows:

- First, we applied a smoothing filter to improve the image and erase some discontinuities in the flagellum. Then, the resulting image was binarized so that we could recover pixels with a high intensity level. The result obtained was an ellipse whose pixels belonged to the trypanosoma’s body. The point ${\vec{w}}_{c}$ was defined as the centroid of this ellipse.
- Once the body was identified, we blocked it by dilating the ellipse which defines it. This ellipse was dilated once more so we could get a mask which allowed us to only see the flagellum. By doing this, the surroundings were eliminated (body and background). We further obtained the histogram of the resulting image. Then, from the histogram, we found the point where the frequency difference between consecutive bins were greater than 40 units, and used this value to binarize the image. The resulting image allowed us to recover the pixels that belong to the flagellum, and the point ${\vec{w}}_{f}$ was defined as the point in the flagellum whose distance to the centroid was the largest.
- Finally, from the ellipse, which defines the body, we found the points belonging to its major axis. We calculated their distance to ${\vec{w}}_{f}$, and and kept the closest point. This point was defined as ${\vec{w}}_{i}$, and it was saved so it could be used as a new point of reference for the next frame—*t*_*i*+1_.

## Results and Discussion

### Parasite trajectory smoothing

**Fig 1:**
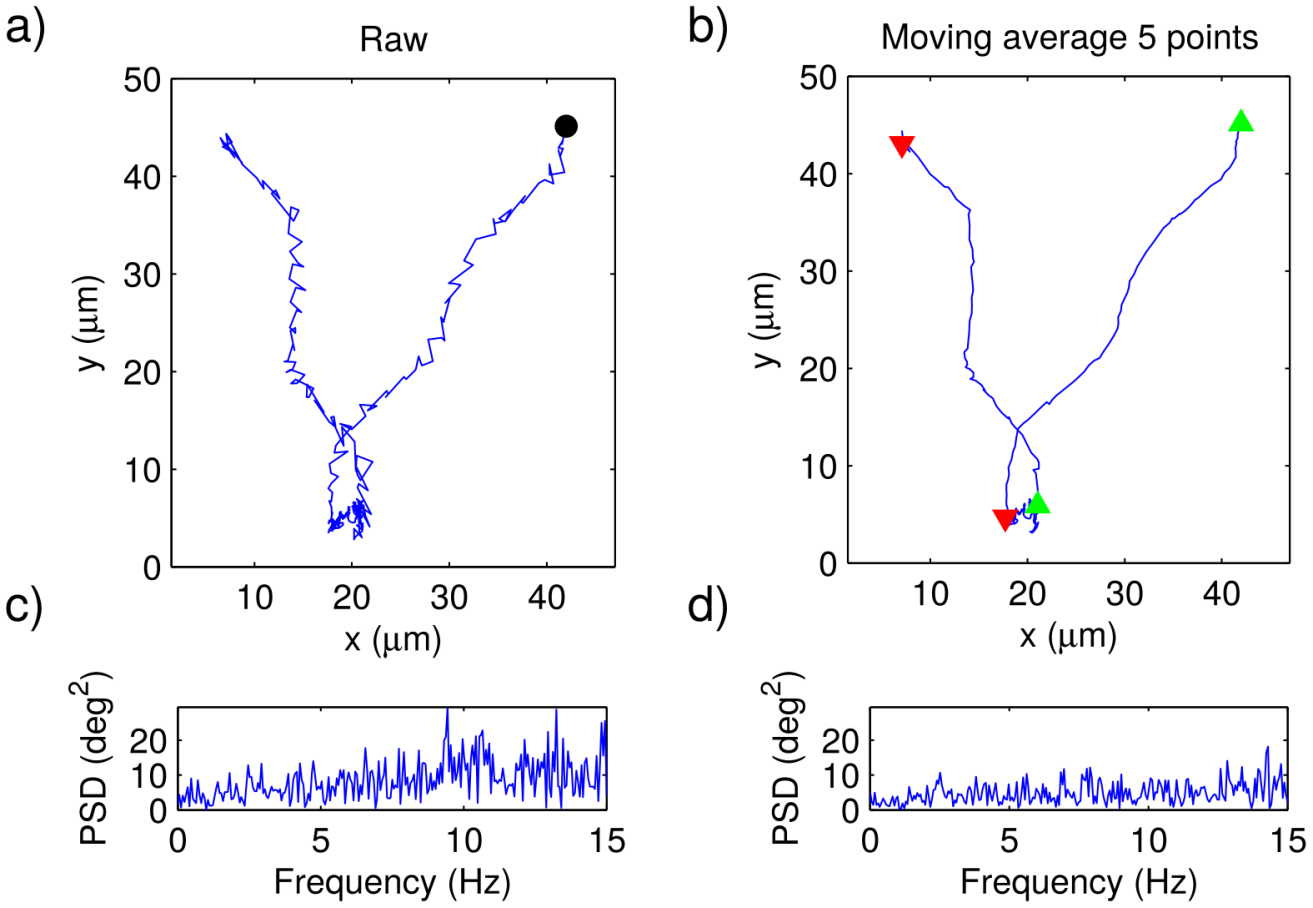
Wild-type parasite trajectories. a) Typical raw trajectory of a wild-type epimastigote (the trajectory initial point is indicated by means of a black dot) and b) the same trajectory after being smoothed by means of a moving average of length 5. The beginning of persistent-motion and tumbling segments are respectively indicated with green and red triangles. In c) and d), the power spectra of the angular changes of direction computed from the trajectories in a) and b), respectively.

**Fig 2:**
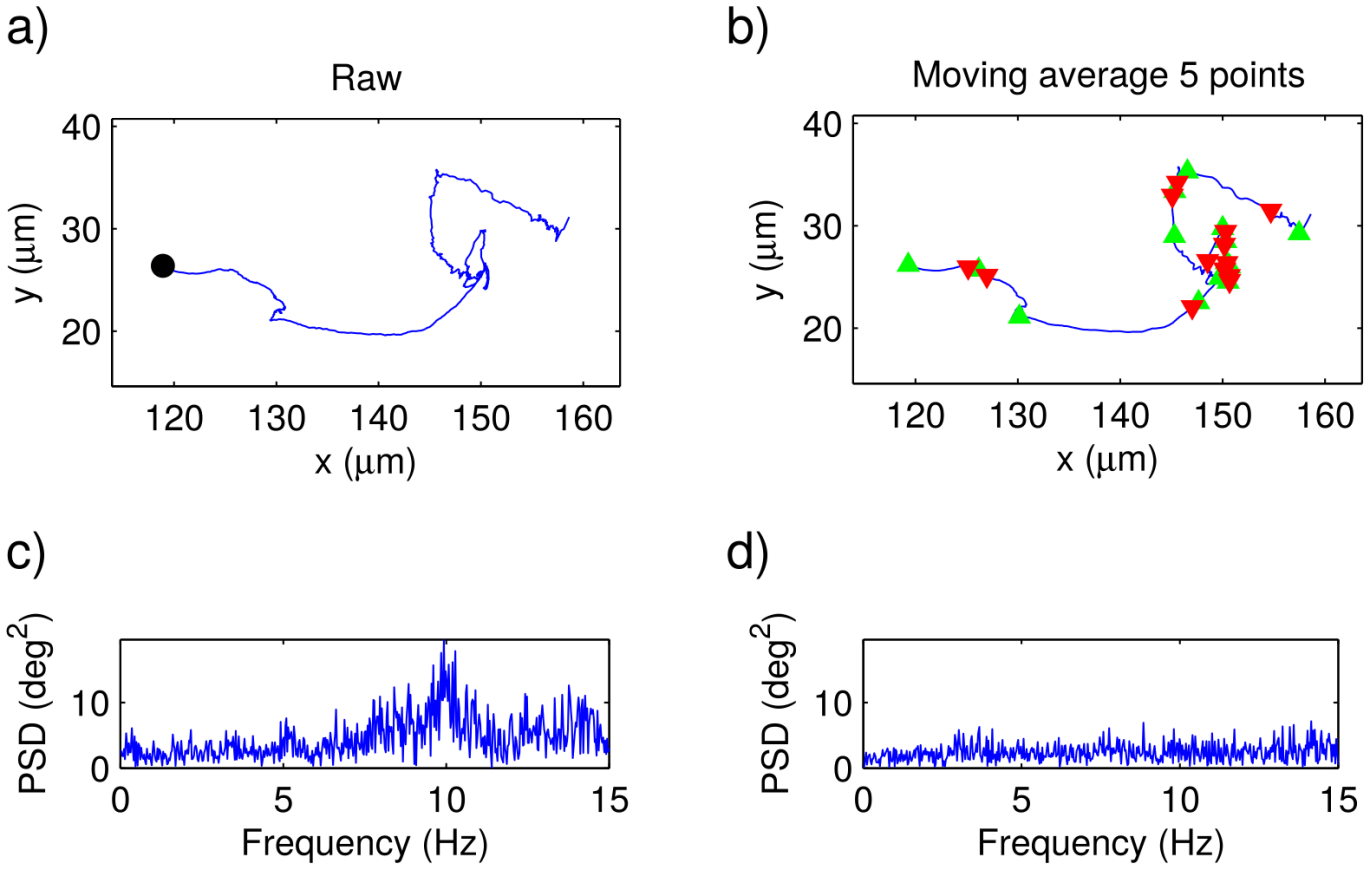
Genetically-modified parasite trajectories. a) Typical raw trajectory of a genetically-modified epimastigote (the trajectory initial point is indicated by means of a black dot) and b) the same trajectory after being smoothed by means of a moving average of length 5. The beginning of persistent-motion and tumbling segments are respectively indicated with green and red triangles. In c) and d), the power spectra of the angular changes of direction computed from the trajectories in a) and b), respectively.

We started by recording, as described in the methodology, parasite trajectories of the two different strains considered in this study. Typical videos for the wild-type and the genetically modified strains are respectively shown in the Supporting Information: S1 Video and S2 Video. We further extracted the trajectory coordinates of all the parasites in each video. In Fig 1a we present a typical raw trajectory for a wild type parasite, and in Fig 2a we do the same for a genetically modified epimastigote. Notice that, in both cases, a regular small-amplitude zig-zag movement can be observed on top of a smother trajectory. We then computed the angular change of direction between consecutive steps, and then calculated the corresponding power spectrum densities (PSDs), one for each trajectory. In Figs 1c and 2c, the PSDs corresponding to the raw trajectories in Figs 1a and 2a are shown. Note that large amplitude peaks appear between about 10 and 15 Hz. By playing the videos in slow motion, it became apparent that the zig-zag movement is due to the flagellum beating. If this is true, according to our results, the flagellum beating frequency should be in the range from 10 to 15 Hz.

**Fig 3:**
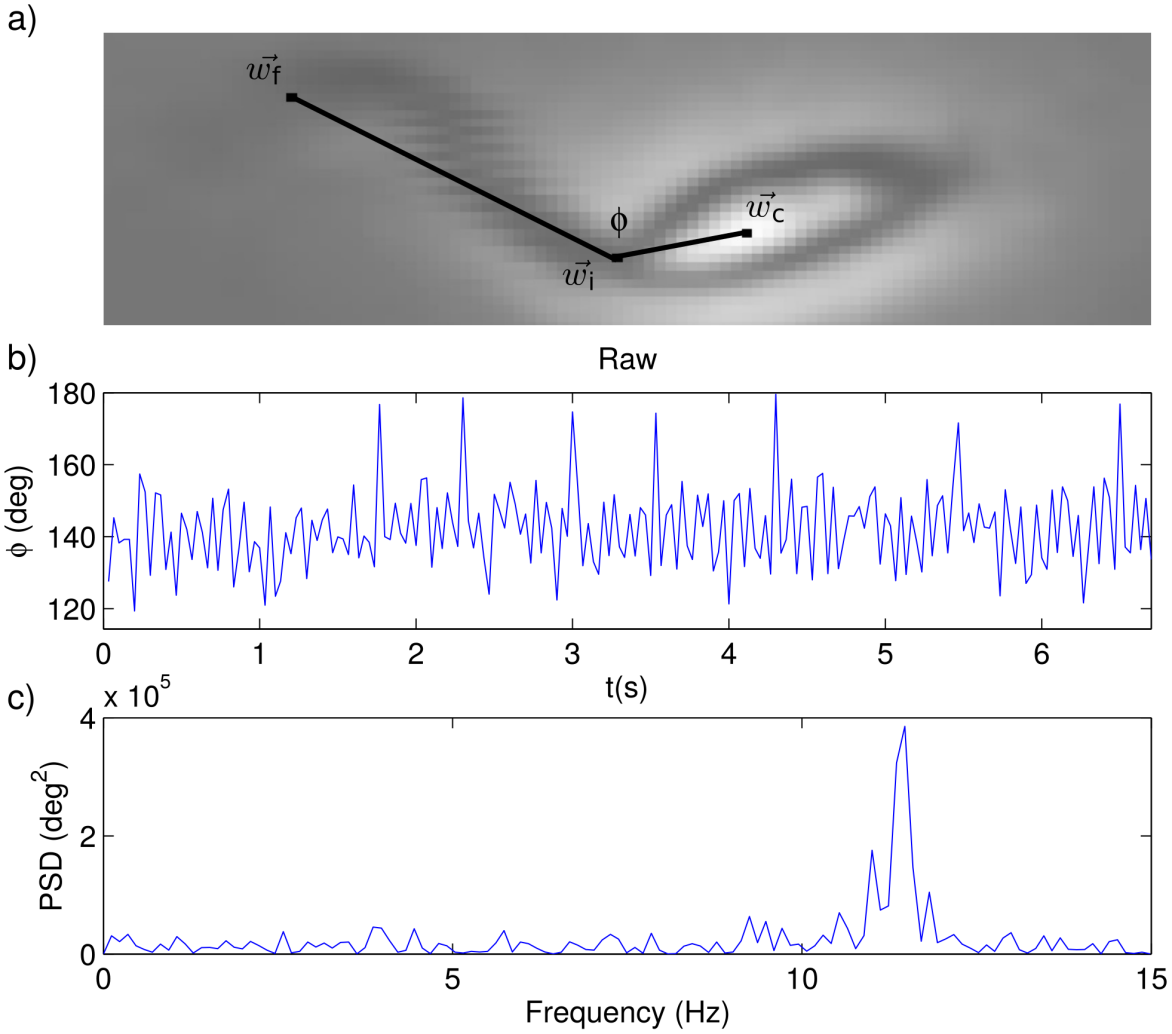
Flagellum beating. a) recorded image of an epimastigote showing the points that identify the centroid of the parasite body (${\vec{w}}_{c}$), the base of the flagellum (${\vec{w}}_{i}$), and its tip (${\vec{w}}_{f}$), all of which are used to compute the angle *ϕ*. b) Time evolution of angle *ϕ* in a typical trajectory. c) Power spectrum of the *ϕ* vs. *t* plot in b).

To corroborate the previous paragraph assertion we analyzed the shape evolution of individual epimastigotes. By following the procedure detailed in the Materials and Methods section, we measured the angle, *ϕ* between a line joining the base and the tip of the flagellum, and the longitudinal axis of the parasite body—see Fig 3a. Then, we followed the time evolution of this angle, repeating the process for 25 parasites. In Fig 3b we present a typical *ϕ* vs. *t* plot, and in Fig 3c we plot the corresponding power spectrum density (PSD). Notice that, as expected, the PSD has notoriously larger peaks in the range from 10 to 15 Hz. Similar results were obtained for all the analized parasites.

Given that we are not interested in the flagellar movements, but only in the long-term displacements they cause, we smoothed the time series corresponding to the *X* and *Y* coordinates of all parasite trajectories by means of a moving average of length 5. By doing so, we expect to filter out all fluctuations whit frequencies larger than 6 Hz. Recall that we recorded parasite trajectories at 30 frames per second.

In Figs 1b and 2b we show the same trajectories as in Figs 1a and 2a, after being smoothed, and in Figs 1d and 2d the corresponding PSDs are plotted. As expected, the zig-zag movements due to the flagellum beating have disapeared, as well as the corresponding peaks in the PSD.

### Persistent and tumbling modes of motion

**Fig 4:**
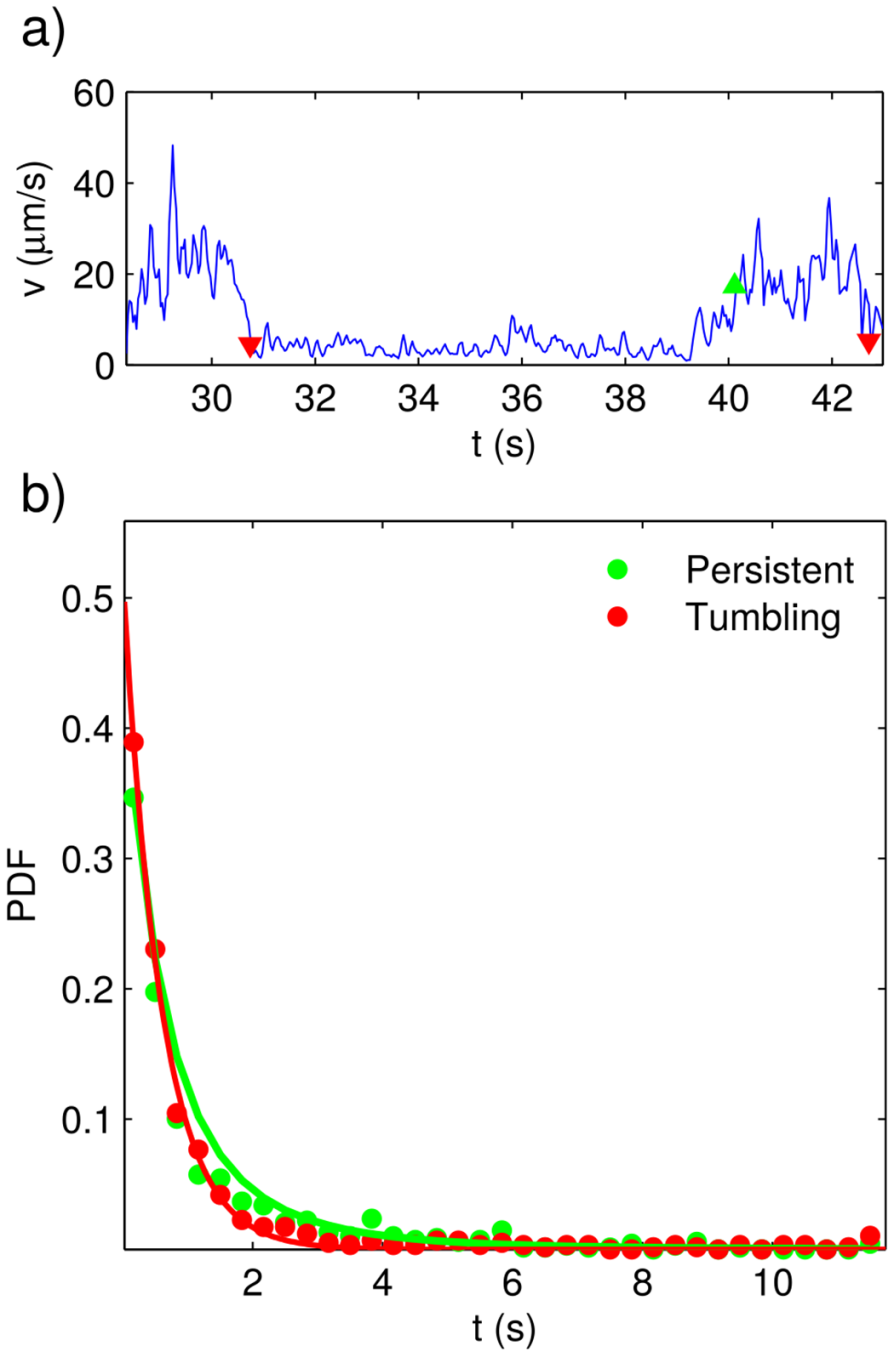
Persistent-motion and tumbling times for the wild-type strain. a) Plot of instantaneous speed vs. time for a typical trajectory. The times at which persistent-motion and tumbling segments begin are respectively indicated with green and red triangles. b) Experimentally determined (dots) and best-fitting distributions (solid lines) for the persistent-motion and tumbling residence times.

**Fig 5:**
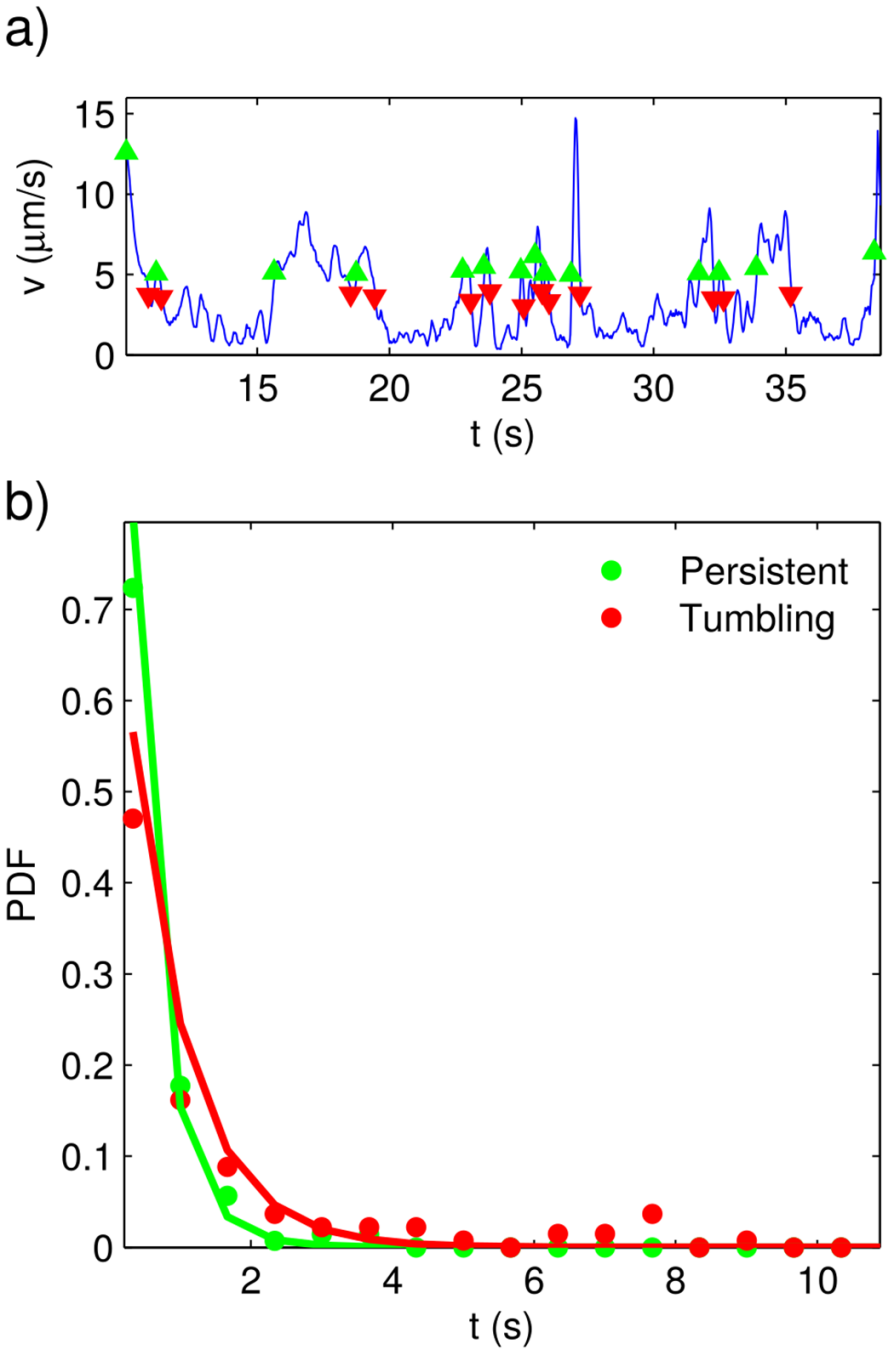
Persistent-motion and tumbling times for the genetically-modified strain. a) Plot of instantaneous speed vs. time for a typical trajectory. The times at which persistent-motion and tumbling segments begin are respectively indicated with green and red triangles. b) Experimentally determined (dots) and best-fitting distributions (solid lines) for the persistent-motion and tumbling residence times.

After smoothing all of the obtained parasite trajectories, we computed for each one of them the step lengths (Δ*r*_*i*_), and the average speed per step *v*_*i*_ = Δ*r*_*i*_/Δ*t*—Δ*t* = 1/30s. In Fig 4a, we can observe a typical speed vs. time plot, corresponding to a wild-type parasite, whereas in Fig 5a the time evolution of the speed of a typical genetically-modified epimastigote is plotted. Notice that, in both cases, the speed fluctuates between two well separated ranges. By visually comparing with the parasite trajectories, we figured out that the two speed ranges correspond to different motility modes which, in accordance to similar studies on *T. brucei* [13, 14], we call persistent and tumbling.

Based on the previous paragraph observations, we programmed and Schmitt trigger to identify the tumbling and persistent-motion segments in each trajectory. This trigger works as follows:

1. Let *v*_*min*_ and *v*_*max*_ be the upper and lower thresholds of the Schmitt trigger. By trial and error we found that *v*_*min*_ = 5*μ*m/*s* and *v*_*max*_ = 12*μ*m/*s* work well for the wild-type strain, while *v*_*min*_ = 4*μ*m/*s* and *v*_*max*_ = 6*μ*m/*s* are adequate for the genetically modified strain.
2. If the initial speed is larger than *v*_*max*_ we say that the parasite mode of motion is persistent. Otherwise, we say that it is tumbling.
3. If at a given time the parasite motility mode is persistent, we consider that it remains being persistent until the parasite speed goes below *v*_*min*_.
4. If at a given time the parasite motility mode is tumbling, we consider that it remains being tumbling until the parasite speed goes above *v*_*max*_.

By means of the previously described Schmitt trigger, we measured the duration of the persistent and tumbling segments in all the obtained trajectories. The points where tumbling and persistent motions begin are respectively illustrated with red and green triangles in Figs 1b, 2b, 4a and 5a. In Figs 4b and 5b, we have plotted the probability density functions (PDFs) for the tumbling and persistent-motion residence-times, respectively estimated from the data measured for the wild-type-strain and the genetically-modified-strain. In the same figures, the corresponding best fitting curves—found via the minimum squared error method—are also plotted. The PDFs for tumbling times was fitted to an exponential distribution,

$$\begin{array}{c} \rho \left(t\right)=\lambda e^{\lambda t}. \end{array}$$ (8)

On the other hand, since the PDFs corresponding to persistent times were heavy trailed, we employed a generalized Pareto distribution to fit them:

$$\begin{array}{c} \rho \left(t\right)=\frac{1}{\sigma }{\left(1+k\frac{t-\mu }{\sigma }\right)}^{-1-\frac{1}{k}}. \end{array}$$ (9)

The best fitting parameter values for the wild-type strain were:

$$\begin{array}{c} \lambda =2.0\;{\text{s}}^{-1},\;k=0.6,\;\sigma =1.2\;\text{s},\;\mu =\frac{1}{6}\;\text{s}. \end{array}$$

Conversely, the best fitting parameter values for the genetically modified strain were:

$$\begin{array}{c} \lambda =1.7\;{\text{s}}^{-1},\;k=0.3,\;\sigma =0.3\;\text{s},\;\mu =\frac{1}{6}\;\text{s}. \end{array}$$

By contrasting Figs 4b and 5b, we can appreciate that the wild-type and genetically-modified PDFs for persistent and tumbling residence times are quite similar. This means that the genetic modifications here considered do not alter the way the parasite shifts between the two different modes of motion.

It is also worth remarking that the tumbling-time PDFs are exponential, whereas the persistent-time PDFs are heavy tailed. The fact that the PDFs of tumbling residence times are exponential means that the shifting from tumbling to persistent motion is completely random, and that the probability of the parasite leaving the tumbling mode of motion is the same at any given time. Conversely, the fact that the persistent-time distributions are heavy tailed means that the longer the parasite remains in persistent motion the smaller the probability that it shifts to tumbling.

### Longitudinal and transverse velocity components

It has been reported [12], and we have observed it as well, that *T. cruzi* epimastigotes move along their longitudinal axis, pulled by their flagellum. From this fact, we assumed that, at a given step, the parasite velocity is parallel to its longitudinal axis, and that the orientation of this axis changes from one step to the next. Based on this supposition, we decided to decompose the epimastigote velocity at every step, into components that are transverse and longitudinal to the velocity of the previous step, in order to study the way the parasite moves.

**Fig 6:**
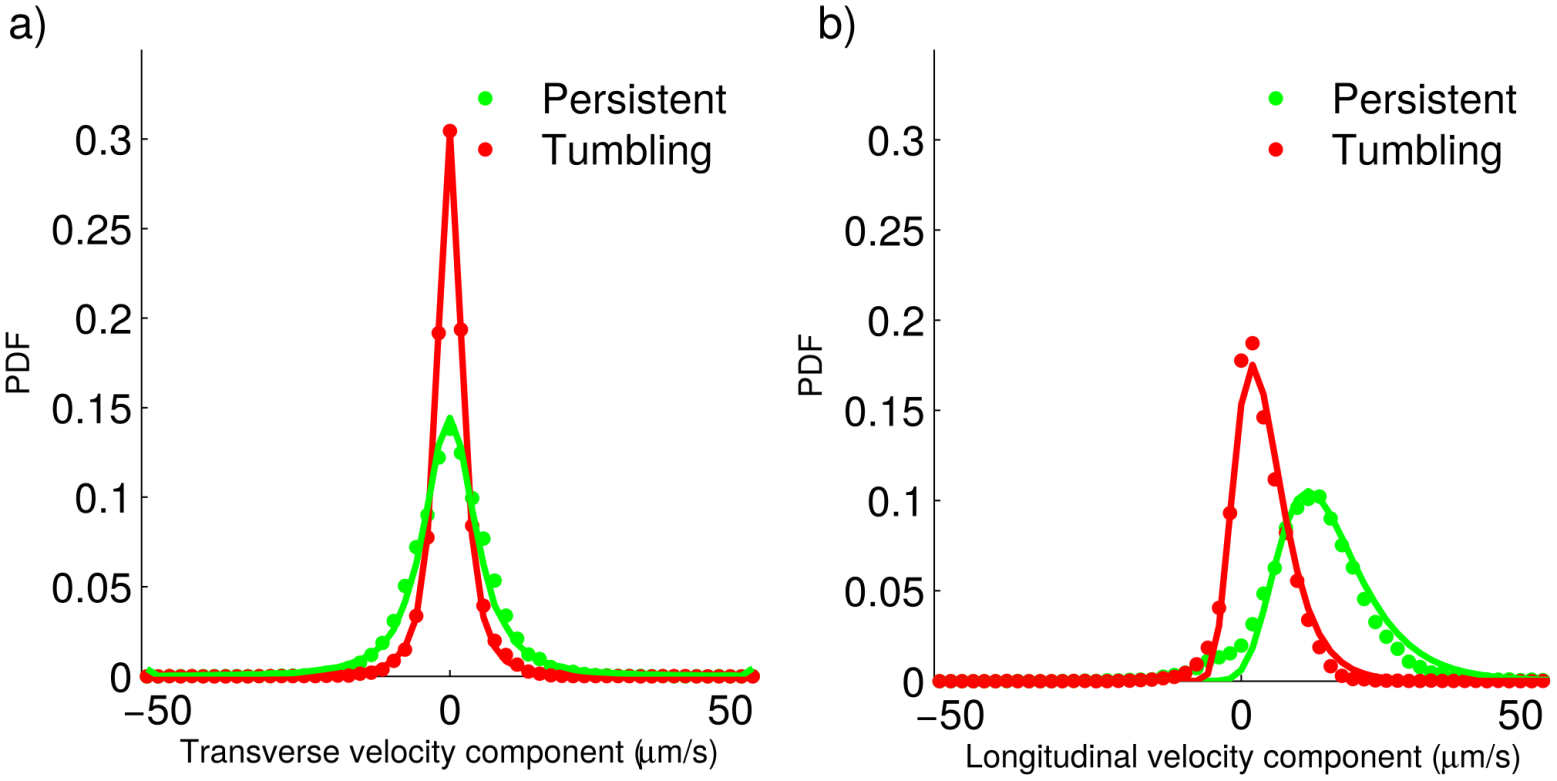
Longitudinal and transverse velocity components for the wild-type strain. a) Experimentally-determined probability density functions (dots) and best fitting distributions (solid lines) for the velocity component transverse to the previous step, during tumbling and persistent motion. b) Experimentally-determined probability density functions (dots) and best fitting distributions (solid lines) for the velocity component longitudinal to the previous step, during tumbling and persistent motion.

**Fig 7:**
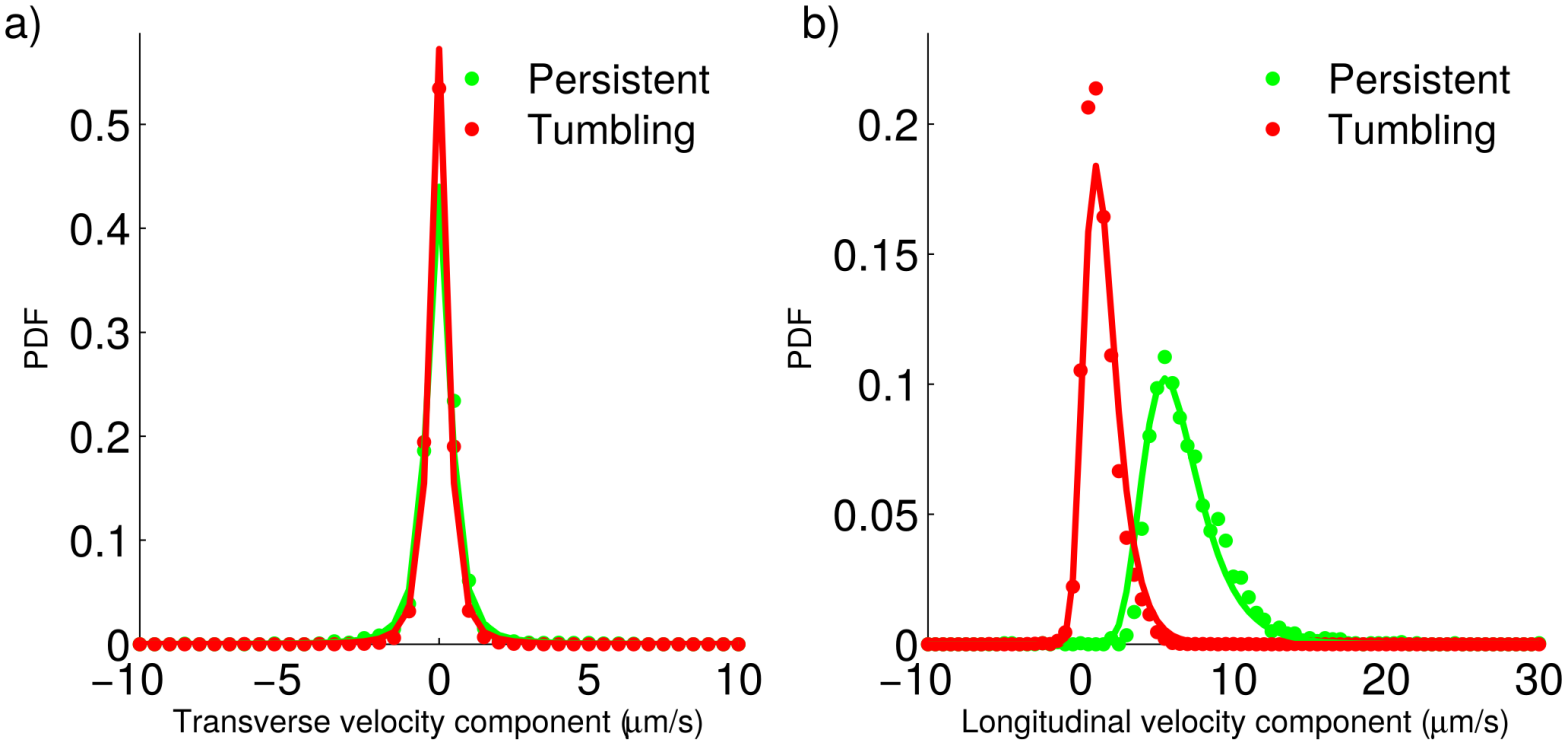
Longitudinal and transverse velocity components for the genetically-modified strain. a) Experimentally-determined probability density functions (dots) and best fitting distributions (solid lines) for the velocity component transverse to the previous step, during tumbling and persistent motion. b) Experimentally-determined probability density functions (dots) and best fitting distributions (solid lines) for the velocity component longitudinal to the previous step, during tumbling and persistent motion.

We computed, by means of Eqs (4) and (5), the longitudinal and transverse components of all the step velocities. We did that for all the recorded trajectories of each strain. Then, we grouped together all the components of each type that correspond to persistent intervals, and repeated the procedure for those corresponding to tumbling intervals. The resulting probability density functions for the wild-type and the genetically modified strains are respectively shown in Figs 6 and 7, together with the corresponding best fitting functions. We found that, in all cases, the PDFs corresponding to the velocity longitudinal components are well fitted by extreme value distributions of the form:

$$\begin{array}{c} \rho \left(x\right)=\frac{e^{\frac{a-x}{b}-e^{\frac{a-x}{b}}}}{b}, \end{array}$$ (10)

while the PDF of transverse velocity components are well fitted by Student’s T distributions with 2 degrees of freedom:

$$\begin{array}{c} \rho \left(x\right)=\frac{1}{\sqrt{8\pi \alpha^{2}}}{\left(1+\frac{{\left(x\right)}^{2}}{2\alpha^{2}}\right)}^{-3/2}. \end{array}$$ (11)

The best fitting parameter values for both strains and for both motility modes are summarized below:

- Wild-type strain, persistent motion,
  $$\begin{array}{c} a=11.0\;\mu \text{m/s},\;b=6.5\;\mu \text{m/s},\;\alpha =2.0\;\mu \text{m/s}. \end{array}$$
- Wild-type strain, tumbling,
  $$\begin{array}{c} a=1.0\;\mu \text{m/s},\;b=3.0\;\mu \text{m/s},\;\alpha =1.5\;\mu \text{m/s}. \end{array}$$
- Genetically-modified strain, persistent motion,
  $$\begin{array}{c} a=6.3\;\mu \text{m/s},\;b=3.0\;\mu \text{m/s},\;\alpha =0.57\;\mu \text{m/s}. \end{array}$$
- Genetically-modified strain, persistent motion,
  $$\begin{array}{c} a=0.95\;\mu \text{m/s},\;b=1.29\;\mu \text{m/s},\;\alpha =0.37\;\mu \text{m/s}. \end{array}$$

Next, we point out some interesting facts regarding the plots in Figs 6 and 7.

- First of all, notice that all the PDFs corresponding to transverse velocity components are symmetric and centered on 0*μ*m/s. Conversely, the PDFs of longitudinal velocity components are asymmetric, the right tail being heavier, and have a single positive mode. All of this is in agreement with our suposition that parasite propulsion mainly affects the longitudinal velocity compoments, while the transverse velocity components can be explained by random factors othen than propulsion due to the flagellum.
- When contrasting the PDFs for the wild-type and the genetically-modified strains, we can see that all the PDFs corresponding to the genetically-modified strain are narrower. Furthermore, in the case of the distributions for the velocity longitudinal components, the modes of the distributions corresponding to the genetically-modified strain are located at smaller values, as compared with those of the wild-type strain. These observations are consistent with the genetically-modified strain moving more slowly than the wild-type strain.

The observations above, together with those of the previous subsection, indicate that the only difference of the genetically-modified and the wild-type strains, regarding motility, is that the first one is slower. Other than that, they seem to move in a very similar way.

### Model development and validation

As previously discussed, our results indicate that *T. cruzi* has two motility modes: persistent and tumbling. At first sight, persistent motion is almost rectilinear. On the other hand, tumbling motion is more complex, but in general it is characterized by having a smaller speed than persistent motion. Their differences can also be appreciated in the probability density functions reported in Figs 6 and 7. Despite their dissimilarity, our analysis suggests that both types of motion can be simulated by adding consecutive steps, with components parallel and perpendicular to the last step that are randomly selected from specific distributions.

Since the parasite is propelled along its longitudinal axis by the flagellum beating, we believe that the so-called longitudinal velocity components can be explained by the parasite propulsion, while transverse velocity components are due to causes other than flagellum-originated propulsion. These suppositions are in agreement with the characteristics of the PDFs reported in Figs 6 and 7. However, to further corroborate that the parasite indeed moves like this, we deviced the model described below, and used it to make predictions to be compared with experimental results:

1. Set the time step Δ*t* = 1/30s.
2. Set the initial trajectory point to (0, 0), as well as the initial simulation time to *t* = 0.
3. Randomly select the initial motility mode: persistent or tumbling, each one having a probability of 0.5.
4. Randomly compute—out of the PDFs in Eqs (8) or (9), and the corresponding parameter values, that depend on the parasite strain and motility mode—the time *T* the simulated parasite will remain in the current motility mode.
5. Calculate the number of steps to be given as the integer part of *T*/Δ*t*.
6. Randomly compute the velocity longitudinal component by means of the PDF in Eq (10) and the corresponding parameter values, depending on the parasite strain. Then, multiply times Δ*t* to get the corresponding displacement.
7. Randomly compute the velocity transverse component by means of the PDF in Eq (11) and the corresponding parameter values, which depend on the parasite strain. Then, multiply times Δ*t* to get the corresponding displacement.
8. Generate the next trajectory point from the results of the two former steps.
9. Update the simulation time *t* := *t* + Δ*t*.
10. Change the parasite motility mode.
11. Iterate from step 4.

**Fig 8:**
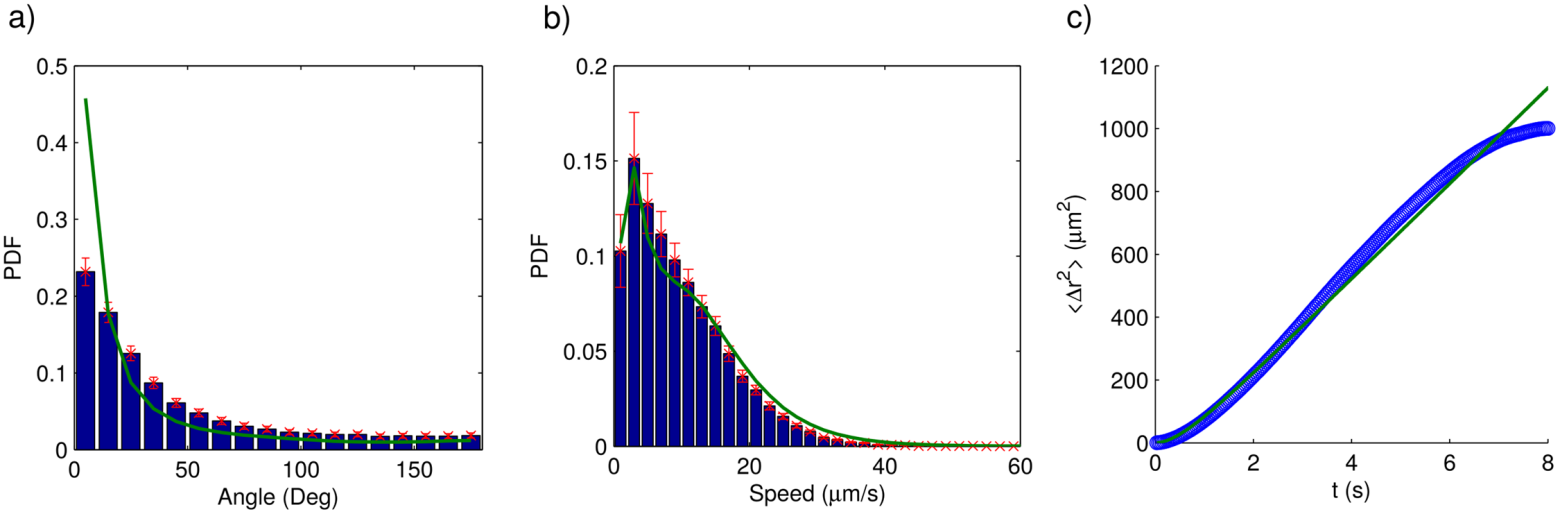
Comparison between model predictions (solid lines) and experimentally determined trajectory characteristics (bars or dots) for the wild-type strain. a) Probability density function for the angular change of direction between consecutive steps. b) Probability density function for the instantaneous speed. c) Mean squared displacement.

**Fig 9:**
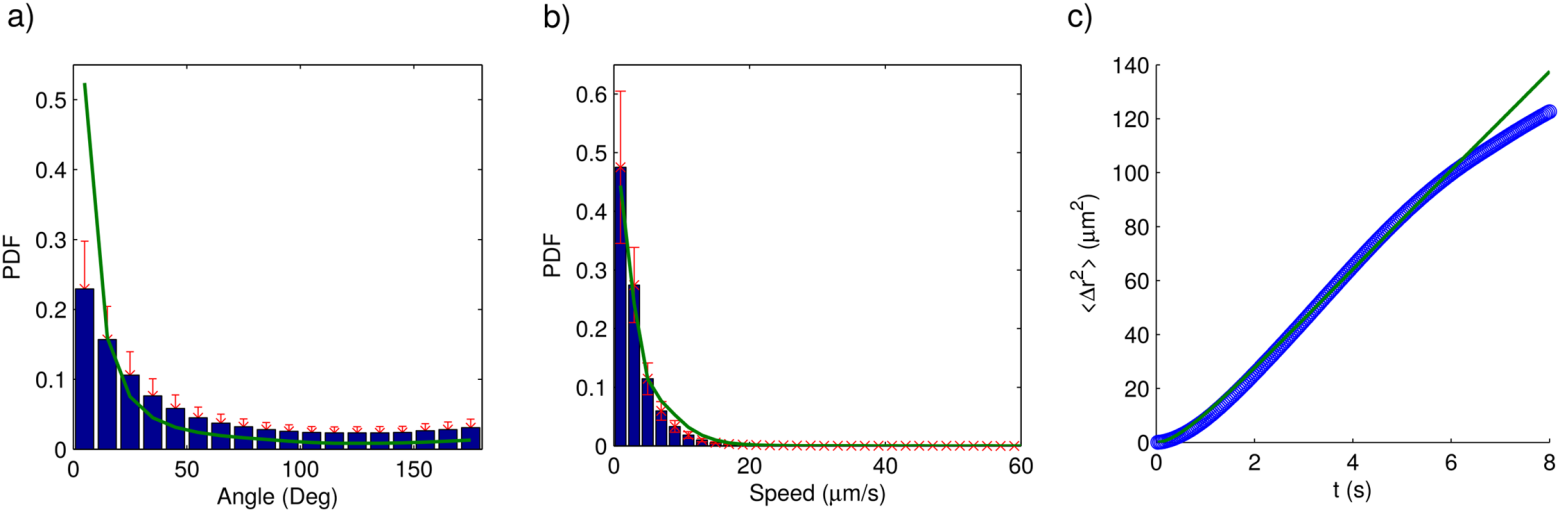
Comparison between model predictions (solid lines) and experimentally determined trajectory characteristics (bars or dots) for the genetically-modified strain. a) Probability density function for the angular change of direction between consecutive steps. b) Probability density function for the instantaneous speed. c) Mean squared displacement.

By using the previously described algorithm we simulated several long parasite trajectories for each parasite strain. Then, we computed the parasite speed in each step, as well as the angular change of direction between consecutive steps—by means of Eq (3). Finally, we estimated the probability density function for each variable. We repeated the whole procedure for the recorded trajectories, and plotted the results in Figs 8 and 9. Note that there is a very good agreement between the experimental and the simulated results. To our consideration, all this validates the assumptions previously introduced model is based on. To further validate the model we calculated the mean squared displacement—see Eq (6)—for both the recorded and the simulated trajectories and plotted the results in Figs 8 and 9. The agreement between the simulated and the experimental curve is also very good in this case. From the above results, we are confident that our model provides a fair description of the way both parasite strains move.

## Concluding Remarks

In this work we have adapted existing methodology [13, 15–19] to study the motility of parasite *T. cruzi* in the epimastigote form. We filmed several epimastigotes of two different strains and, by means digital image-processing techniques, we obtained their trajectories, and analyzed them by extracting different characteristics like: instantaneous velocity and angular change of direction.

A visual analysis of the speed vs. time plots revealed the existence of two modes of motion for this parasite. In agreement with previous studies on *T. brucei* [13, 14], we called them tumbling and persistent. After computing the residence times in each of these motility modes for several parasites, we found that probability density function (PDF) of tumbling times can be fitted by an exponential distribution, whereas the PDF of persistent-motion times is heavy tailed. This means that the propensity (or probability per unit time) of the parasite shifting from tumbling to persistent motion is constant, but the propensity of the parasite shifting from persistent motion to tumbling is a decreasing function of time. In other words, epimastigotes have some kind of memory when their motion mode is persistent because, the longer they remain in this state, the less likely that they shift to tumbling.

Interestingly, switching between different modes of motion has been observed in organisms whose sizes differ by many orders of magnitude. For instance, *E. coli* changes the orientation of one or more of its flagella between clockwise and anticlockwise to achieve a run-and-tumble-like motion [15, 17]. Generally, this behavior is associated to chemotaxis or some other types of taxis. Thus, although it hasn’t been experimentally proved, this finding suggests that *T. cruzi* epimastigotes experience some kind of taxis. Given that epimastigotes are primarily found in the triatome gut, where they have to attach to epithelial cells to reproduce, they might be sensitive to some chimioattractant secreted by these cells.

During persistent motion the parasite moves in a quasi rectilinear fashion. On the other hand, a visual inspection of the videos reveals that tumbling motion is more complex and difficult to describe. The most noticeable characteristic being that epimastigote speed during tumbling is noticeable smaller than during persistent motion. Despite these intricacies, we found that decomposing trajectory steps in components longitudinal and traversal to the previous step, allows a good description of the parasite motility. Indeed, we were able to develop a model to simulate parasite trajectories that accurately reproduced characteristics of the experimental trajectories such as: the probability density functions for the speed and the angular change of direction between consecutive steps, as well as the mean squared displacement.

The model here proposed feeds on the PDFs of longitudinal and transverse velocity components, experimentally obtained for both the tumbling and persistent modes of motion. From the way it simulates parasite trajectories, this model can be regarded as a more detailed instance of the velocity jump process originally introduced in [25]. Despite its capability to reproduce parasite trajectories, the model is phenomenonlogic and thus it cannot explain the origin of various interesting features of *T. cruzi* epimastigote motility. For example, the fact that the PDFs of longitudinal velocity components are asymmetric and it’s mode is larger than zero can be explained by flagella propulsion. However, it’s not clear why the PDFs of transverse velocity components are heavy tailed.

Finally, by comparing the analysis we performed on the wild type strain and on a genetically modified strain that over-expresses both isoforms of protein LYT1, we found that both strains essentially move in the same way, except that the genetically modified strain does it more slowly. This agrees with the above discussed possible downstream effects of LYT1 over-expression, with regards to *T. cruzi* motility. Nonetheless, more work is necessary to demonstrate this last assertion.

## Supporting Information

S1 Video Wild-type *T. cruzi* epimastigote trajectories. Typical video showing the parasite movements.
(MP4)

S2 Video Genetically-modified *T. cruzi* epimastigote trajectories. Typical video showing the parasite movements.
(MP4)
